# Patient Satisfaction with Psychiatric Outpatient Care at University of Gondar Specialized Hospital: A Cross-Sectional Survey

**DOI:** 10.1155/2019/5076750

**Published:** 2019-04-01

**Authors:** Nigist Alemayehu Woldekidan, Begashaw Melaku Gebresillassie, Rediet Hagos Alem, Biruk Fentahun Gezu, Ousman Abubeker Abdela, Assefa Belay Asrie

**Affiliations:** ^1^Department of Clinical Pharmacy, School of Pharmacy, College of Medicine & Health Sciences, University of Gondar, Gondar, Ethiopia; ^2^Department of Pharmacology, School of Pharmacy, College of Medicine & Health Sciences, University of Gondar, Gondar, Ethiopia

## Abstract

**Background:**

Patient satisfaction is an imperative and commonly used indicator for measuring the quality of healthcare. Patient satisfaction with psychiatry services is an important construct, which influences multiple areas including treatment adherence and outcome. The aim of the present study was to assess the level of patient satisfaction and determine associated factors with psychiatric outpatient care.

**Method:**

An institution-based cross-sectional study was conducted from April 15 to May 15, 2017. A total of 250 psychiatric patients visiting psychiatric outpatient care in University of Gondar Specialized Hospital during the study period were included in the study. Data were collected using structured questionnaires and entered to and analyzed using Statistical Packages for Social Sciences (SPSS) version 20. Descriptive statistics and one-way ANOVA with post hoc test were used to determine the characteristics of the participants and examine the difference among different variables. P value<0.05 and confidence interval (CI) of 95% were used as cut-off points for determining statistical significance.

**Results:**

During the one-month data collection period, 250 participants (92.593% response rate) were included in the analysis. Majority (133 (53.2%)) of them were males and cannot read and write (107 (42.8%)). Majority (194 (77.6%)) of study participants were satisfied with the outpatient care. The overall level of satisfaction among participants was good with a mean satisfaction score of 3.87. Majority (173 (69.2%)) of the participants claimed that health professionals working in outpatient care did not provide adequate information about payment for services. Statistically significant satisfaction difference with respect to the psychiatric outpatient care was found in the type of mental illness (t=2.224, P=0.043) and the participants' employment status (t=2.981, P=0.003).

**Conclusion:**

In general, the overall participants' satisfaction towards outpatient care was high. Statistically significant satisfaction difference with respect to the psychiatric outpatient care was found in the type of mental illness and the participants' employment status. Regular service evaluation is important to improve patient satisfaction and further research is needed to investigate why satisfaction difference exists among different types of mental illnesses.

## 1. Background

Clinicians and healthcare manager's perception agreed that improving quality of care is getting main concern and persistent challenge [1]. Hence, patient satisfaction is usually used as an indicator for measuring the quality of healthcare. It affects medical management condition, patient maintenance, and clinical outcomes. It also influences the timely, efficient, and patient-centered delivery of quality health service [2]. High treatment failure rates and poor medication adherence in psychiatry could be turned around by focusing on factors that affect patient satisfaction and commitment to therapy [3].

In recent decades' patients' insight into healthcare has gained increasing attention in mental health services [4, 5]. Symptomatic evaluation does not reflect all of the factors that patients consider to be important in their life and that patient's views should supplement the usual indicators of quality in mental healthcare [6, 7]. The most study conducted in this area showed the highest level of satisfaction to psychiatric service, 90% in Ireland [8], 91.9% in South Africa [9], and 83% in Nigeria [10]. However, a low satisfaction level was reported from studies conducted in India, 57%, and Ethiopia, 61.1% [11, 12].

Studies also indicate a variety of factors can affect patient satisfaction. A new meta-analysis identified dozens of patients provide related factors that affect patient satisfaction and commitment in psychiatry, many of which are related to patients' self- efficient and knowledge and the quality of their relationship with their healthcare provider. Barriers to the dedication, which directly related to patient satisfaction, were identified [13]. The previous study also indicated that patient satisfaction can also have affected by many factors such as patients' demographics and attitude towards the service [12, 14], duration of disease, diagnosis, treatment duration, and patients' expectation of service [14–17]. Undeniably patient satisfaction studies do yield valuable information about the accessibility of quality healthcare as well as concerning true or fake patient participation, adequacy of information, appropriate allocation of resources, and interest of health policy decision-makers.

Most of the limited researches in the area of psychiatry care in Ethiopia have been focused on health institution-based prescription pattern assessments and some basic researches. To the best of literature searches done, there were little studies which assessed patients' satisfaction towards the services they get in Ethiopia. However, there have been reports of varying complaints of poor satisfaction about the service in hospitals and health centers with no empirical evidence to support them. The aim of this study was to help narrow the information gap in this respect by documenting the satisfaction level of patients in psychiatric outpatient care, University of Gondar Specialized Hospital (UOGSH). The result will also have implication for health professionals working in the psychiatry clinic to improve the quality of services they provide. Together with this, it is crucial to provide recommendations for concerned stakeholders including pharmacists, physicians, psychiatry nurses, clients, hospital, and health administrations.

## 2. Methods

### 2.1. Study Area and Study Period

The study was conducted on satisfaction and associated factors in outpatient psychiatric care among psychiatric patients at University of Gondar Specialized Hospital. University of Gondar Specialized Hospital is one of the oldest teaching hospitals in the country, located 738 km northwest of Addis Ababa which is a capital city of Ethiopia. According to 2016 University of Gondar Specialized Hospital Statistics and Information Office: Annual Report on Health Services and Employees, the hospital contains more than 400 beds and provides its services in various departments including pediatrics, surgery, gynecology, psychiatry, dermatology, dentistry, ophthalmology, pharmacy (outpatient, inpatient, antiretroviral, and emergency), medical laboratory, and others. Particularly the psychiatry unit contains 21 beds and on average 300 new patients are admitted every month. It was conducted from April 15 to May 15, 2017.

### 2.2. Study Design

An institution-based cross-sectional study was conducted in an outpatient psychiatric care unit of UOGSH.

### 2.3. Population

The source population includes clients who use outpatient psychiatric care unit, at UOGSH, northwestern Ethiopia, whereas the study was conducted on clients attending outpatient psychiatric care unit during the study period.

### 2.4. Inclusion and Exclusion Criteria

Patients (18 years and above) who received treatment for at least 6 months from the outpatient psychiatry clinic were included in the study, whereas patients with disabilities which hinder filling out questionnaires or interviewing with investigators were excluded.

### 2.5. Sample Size Determination

A census was conducted on patients visiting psychiatry clinic during the study period.

### 2.6. Study Variables

Independent variables include age, sex, marital status, religion, educational status, ethnicity, income, occupational status, the area of residence, type of mental illness, duration of illness, and a number of medications. On the other hand, level of satisfaction with the services was the dependent variable.

### 2.7. Data Collection and Management

The data was collected by three investigators through a self-administered questionnaire and face to face interview for those individuals unable to read and write. The data collection instrument was adapted from prior studies and consist of sections focusing on sociodemographic characteristics and satisfaction with the psychiatric outpatient care [12, 18]. To assess patient satisfaction, we have used a standardized satisfaction measurement tool developed for low income countries [19] and contained five-point Likert scale items, on scale “1” stood for rating of the item as “poor” while “2”, “3”, “4”, and “5” stood for “fair”, “good”, “very good”, and “excellent”, respectively. The mean level of satisfaction was calculated by averaging their ratings for the parameters of measuring satisfaction. If the patient scored 3 and above, the client high level of satisfaction was interpreted, whereas if he/she scored below 3 he/she has a low level of satisfaction. The data was collected from April 15 to May 15, 2017.

### 2.8. Data Quality Assurance

A standardized tool was used to assess the patient satisfaction 17, prepared in English. Then, it was translated to Amharic local language and then back to English in order to ensure that the translated version gives the proper meaning. It was also pretested on 20 patients who were not included in the final analysis and relevant modifications were instituted before the commencement of actual data collection. The investigators who collected the data were properly trained on the instrument and ways of approaching the patients and securing their permission for an interview prior to the data collection process.

### 2.9. Data Analysis

The data collected using quantitative method was entered to and analyzed using Statistical Packages for Social Sciences (SPSS) version 20 statistical software. Frequencies, percentages, one-way ANOVA with post hoc test were used to examine difference among different variables. P value <0.05 and confidence interval (CI) of 95% were used as cut points for determining statistical significance.

### 2.10. Ethical Consideration

The present study was conducted after ethical clearance was gained from research and ethics review committee of School of Pharmacy and the Clinical Directorate of University of Gondar Specialized Hospital. All participants were provided with oral explanations on the purpose of the study and again orally asked for their consent to participate in the study. They were also informed that participation was voluntary and they could withdraw from the study at any stage if they desired. Information obtained from the questionnaires was kept confidential. In addition, patient identifiers were not used and the data collected was used by the investigators only for the purpose of the study.

## 3. Results

### 3.1. Sociodemographic Characteristic

In the present study out of 270 patients who were interviewed 250 were included in the analysis, and 20 encounters were excluded due to incompleteness making the response rate 92.59%. From those included in the study 143 (45.2%) of the participants were in the age range of 18-30 years and cannot read and write 107 (42.8%). Nearly two-thirds 146 (58.4%) of the participants were married or ever married and live in urban area 143 (57.2%). Of the total participants, 189 (75.6%) were Orthodox and 246 (98.4%) were Amhara in ethnicity. Majority of the participants were either self-employed or unemployed and their monthly income is less than 500 Ethiopian Birr (ETB) (Table 1).

### 3.2. Clinical and Patient-Related Factors

Regarding clinical characteristics of the respondents, 130 (52%) had schizophrenia, 77 (30.8%) had major depression, 28 (11.2%) had bipolar disorder, and 15 (6%) had anxiety disorders. More than two-thirds (168 (67.2%)) of the respondents had the duration of illness between 1 year and 5 years. Half 126 (50.4%) of them had been taking antipsychotics, followed by antidepressant 63 (25.2%), and the combination of both antipsychotic and antidepressant 53 (21.2%).

### 3.3. Patients' Satisfaction towards the Psychiatric Outpatient CARE

The overall satisfaction was described by parameters for psychiatric outpatient care that were used to measure the level of patient satisfaction. Participants were considered as highly satisfied with the general services if they scored a mean value higher than 3 (a score considered as “good”). Majority (194 (77.6%)) of study participants were satisfied with the outpatient care. The overall level of satisfaction among participants was good with a mean satisfaction score of 3.87.

Among the parameters the participants were unsatisfied with the service related to opportunity for follow-up with the same health worker with the mean satisfaction score of 1.99. Most of the participants also claimed that health professionals working in psychiatric outpatient care did not tell adequate information about payment for services with a mean satisfaction score of 2.55. They had also relatively low satisfaction regarding finance to come and get treatment, and the possibility of referral to a specialist with a mean satisfaction score of 2.57 and 2.58, respectively. On the other hand, they have higher satisfaction score with the provision of helpful advice; time is given for discussion with health professionals and about the location of an outpatient clinic with a mean satisfaction score of 4.63, 4.40, and 4.60, respectively (Table 2).

### 3.4. Satisfaction Level Difference towards Psychiatric Outpatient Care

The difference in the mean satisfaction level of respondents involved in the study was checked with respect to sociodemographic characteristics. Based on one-way ANOVA test performed on sociodemographic variables, statistically significant satisfaction difference with respect to the psychiatric outpatient care was found in the type of mental illness (P=0.043) and the participants' employment status (P=0.003) (Table 3).

## 4. Discussion

The present study examined the satisfaction level of patients attending the psychiatric outpatient care at one of the tertiary level teaching hospital where the standards of care are expected to be high. Studies assessing patients' satisfaction level are vital in obtaining a comprehensive understanding of the patients need and their opinion of the service received. This will help to mitigate the discrepancy between what the patients need and what they really get [20].

Majority (194 (77.6%)) of study participants were satisfied with the outpatient care. The overall level of satisfaction was good with a mean satisfaction score of 3.87. This result was higher than previous study conducted in Ethiopia which was 61.2%. On the other hand, comparable findings were reported from India, Norway, Nigeria, Sweden, and Copenhagen with 57%, 75%, 83% 77%, and 80.4% satisfaction level, respectively [10–12, 21–23]. However, the magnitude of satisfaction found in the present study was slightly lower than those of the studies conducted in Ireland, South Africa, India, and Pakistan, which reported the prevalence rate of 90%, 87.25%, 92.8, and 90.7%, respectively [8, 9, 24, 25].

The majority (92.6%) of the study participants were satisfied with the helpful advice they get from the psychiatry nurses with a mean satisfaction score of 4.63. This was much higher than that reported from Dessie, which was 70.3% for a similar question [12]. The reason attributed to this could be the variation of staff profile and years of experience. Similarly, a majority (92%) of participants were satisfied regarding the location and cleanness of the outpatient care. This was also higher than that reported from Pakistan, which was (79.8%) [26]. Participants were unsatisfied with the service related to the opportunity for follow-up with the same health worker with the mean satisfaction score of 1.99. During patient visit variation of one healthcare provider could have no significant effect on the patient outcome but when there are multiple changes, the patient outcome could be markedly affected [27]. Variation of healthcare provider during various visits can confuse a patient knowing who to contact during need for help. Together with this, the majority of the patients are also unwilling to closely approach and tell details about their life for the changing healthcare provider. This might be very important to ensure appropriate diagnosis and follow-up.

Half (51%) of the participants claimed that health professionals working in psychiatric outpatient care did not tell adequate information about payment for services with a mean satisfaction score of 2.55. This finding was consistent with the study done in Switzerland (49%) [28] and Dessie (42.2%) [12]. The type and extent of information provided as well as the type of communication maintained between patient and healthcare provider may have an impact on the whole process of care [19]. Although most of the time patients forget or ignore advice and relevant information provided, it can be improved by the health providers' commitment.

In the present study, one-way ANOVA analysis was performed on sociodemographic characteristics and statistically significant satisfaction differences were found among different type of mental illnesses and employment status of the patient. On post hoc analysis significantly higher level of satisfaction was reported from patients with schizophrenia compared to patients with bipolar disorder. This could indicate that the psychiatric outpatient care majorly focused on the frequently identified mental illness that needs acute intervention.

## 5. Limitation of the Study

The limitation of the present study including each and every service provided to the patients in the psychiatric outpatient care was not assessed in detail. This might interfere with the ability of this study to assess the level of satisfaction with regard to the kind of services received. Furthermore, we only included patients who attended the outpatient care and those who had defaulted on their appointments were excluded. This subgroup would have had higher levels of dissatisfaction if they were included.

## 6. Conclusion

This is the first attempt to assess participants' satisfaction with the services of psychiatric outpatient care in UOGSH. In general, participants' satisfaction from the outpatient services is good. The highest satisfaction was found with regard to receiving helpful advice and explanation from the healthcare provider, whereas the lowest satisfaction was observed with regard to lack of opportunity for follow-up with the same healthcare provider. Satisfaction of the participants towards the service was low in those who claimed that healthcare providers working in the outpatient care did not provide adequate information about payment for services. Hence, regular service evaluation is important to improve patient satisfaction and further research is needed to investigate why satisfaction difference exist among different types of mental illnesses.

## Figures and Tables

**Table 1:** Distribution of participants by socio-demographic characteristics, UOGSH, 2017.

Variables (N=250)	Category	Frequency (%)
Age (in years)	18-30	113(42.2)
	31-40	99(39.6)
	41-50	23(9.2)
	>50	15(6.0)

Sex	Male	133(53.2)
	Female	117(46.8)

Religion	Orthodox	189(75.6)
	Muslim	60(24.0)
	Protestant	1(0.4)

Ethnicity	Amhara	246(98.4)
	Tigray	4(1.6)

Marital status	Single	104(41.6)
	Married/ever married	146(58.4)

Education status	Cannot read and write	107(42.8)
	Primary school (1-8)	46(18.4)
	Secondary school (9-10)	41(16.4)
	College and above	56(22.4)

Employment status	Government employed	33(13.2)
	Self-employed	108(43.2)
	Unemployed	109(43.6)

Area of residence	Urban	143(57.2)
	Rural	107(42.8)

Monthly income (in ETB)	<500	186(74.4)
	500-1499	38(15.2)
	1500-2499	16(6.4)
	>2500	10(4.0)

Distance from hospital	<20	87(34.8)
	21-40	76(30.4)
	41-60	60(24.0)
	>61	27(10.8)

ETB: Ethiopian Birr.

**Table 2:** Patients' satisfaction towards psychiatric outpatient care, UOGSH, 2017.

Variables	Poor, Fair n(%)	Good, Very good, Excellent n(%)	Mean satisfaction level
The health worker treated me with courtesy	19(7.6)	231(92.4)	4.56

The health worker listened to me carefully	15(6.0)	235(94.0)	4.59

The health worker explained me things in a way I understood	15(6.0)	235(94.0)	4.57

Location of the outpatient service is acceptable	9(3.6)	241(96.4)	4.60

The health facility was clean	14(5.6)	236(94.4)	4.60

The waiting area was clean	16(6.4)	234(93.6)	4.59

The latrine was clean	45(18.0)	205(82.0)	4.38

The waiting time was acceptable	29(11.6)	221(88.4)	4.31

I have enough time to discuss with a health worker	22(8.8)	228(91.2)	4.40

I was given information in a way I understood	15(6.0)	235(94.0)	4.53

I received helpful advice	12(4.8)	238(95.2)	4.63

The administrative staff treated me with courtesy and respect	40(16.0)	210(84.0)	4.39

My privacy is respected	35(14.0)	215(86.0)	4.37

I have the opportunity to follow up with the same health worker	228(91.2)	22(8.8)	1.99

Adequate information provided about payment for services	173(69.2)	77(30.8)	2.55

My personal information is kept confidential	45(18.0)	205(82.0)	4.26

Referral to a specialist is possible	176(70.4)	74(29.6)	2.58

The treatment is effective at decreasing symptoms	18(7.2)	232(92.8)	4.50

The treatment is effective at decreasing relapses	22(8.8)	228(91.2)	4.43

The treatment helped me to improve my income	136(54.4)	114(45.6)	3.14

I can get a health worker's help any time I need	34(13.6)	216(86.4)	4.07

It was easy to come to the hospital	141(56.4)	109(43.6)	2.90

I had enough time to come to the hospital	143(57.2)	107(42.8)	2.88

I had enough money to come and get treatment	168(67.2)	82(32.8)	2.57

I would advise my family to come to the hospital	103(41.2)	147(58.8)	3.27

The service is effective at helping with economic problems	125(50.0)	125(50.0)	2.99

**Table 3:** Test of statistical significance (one-way ANOVA test) of the variation in the mean satisfaction level of participants by sociodemographic characteristics and other factors suspected to affect patient satisfaction, UOGSH, 2017.

Variables		Overall satisfaction	
		Mean(SD)	P- Value
Age (in years)	18-30	3.83(0.578)	0.507
	31-40	3.93(0.387)	
	41-50	3.88(0.400)	
	>50	3.82(0.511)	

Education status	Cannot read and write	3.85(0.470)	0.213
	Primary school (1-8)	3.97(0.420)	
	Secondary school (9-10)	3.77(0.527)	
	Tertiary	3.90(0.545)	

Employment status	Government employed	3.95(0.515)	0.003*∗*
	Self-employed	3.97(0.416)	
	Unemployed	3.75(0.528)	

Type of mental illness	Schizophrenia	3.89(0.450)	0.043*∗*
	MDD	3.87(0.526)	
	Bipolar disorder	3.65(0.575)	
	Anxiety disorder	4.02(0.432)	

Duration of illness	<5	3.88(0.504)	0.602
	6-10	3.90(0.434)	
	11-15	3.83(0.593)	
	>15	3.69(0.469)	

Classes of drugs indicated	Antipsychotics	3.89(0.466)	0.443
	Antidepressants	3.88(0.526)	
	Combination of both antipsychotics and antidepressants	3.79(0.516)	
	Others*∗∗*	4.04(0.372)	

Monthly income (in ETB)	<500	3.83(0.499)	0.117
	500-1499	3.96(0.370)	
	1500-2499	4.08(0.332)	
	>2500	3.95(0.792)	

*∗*P value less than 0.05, *∗∗*carbamazepine, sodium valproate, diazepam, and MDD: major depressive disorder.

## Data Availability

The materials and data of this study are available from the corresponding author upon request.
